# Vehicle-Type Recognition Method for Images Based on Improved Faster R-CNN Model

**DOI:** 10.3390/s24082650

**Published:** 2024-04-21

**Authors:** Tong Bai, Jiasai Luo, Sen Zhou, Yi Lu, Yuanfa Wang

**Affiliations:** 1 School of Optoelectronic Engineering, Chongqing University of Posts and Telecommunications, Chongqing 400065, China; baitong@cqupt.edu.cn (T.B.); luyileo@cqupt.edu.cn (Y.L.); wangyf@cqupt.edu.cn (Y.W.); 2 Chongqing Academy of Metrology and Quality Inspection, Chongqing 401121, China; zhousen@cqu.edu.com

**Keywords:** vehicle-type recognition, faster R-CNN, contextual features, bounding box

## Abstract

The rapid increase in the number of vehicles has led to increasing traffic congestion, traffic accidents, and motor vehicle crime rates. The management of various parking lots has also become increasingly challenging. Vehicle-type recognition technology can reduce the workload of humans in vehicle management operations. Therefore, the application of image technology for vehicle-type recognition is of great significance for integrated traffic management. In this paper, an improved faster region with convolutional neural network features (Faster R-CNN) model was proposed for vehicle-type recognition. Firstly, the output features of different convolution layers were combined to improve the recognition accuracy. Then, the average precision (AP) of the recognition model was improved through the contextual features of the original image and the object bounding box optimization strategy. Finally, the comparison experiment used the vehicle image dataset of three vehicle types, including cars, sports utility vehicles (SUVs), and vans. The experimental results show that the improved recognition model can effectively identify vehicle types in the images. The AP of the three vehicle types is 83.2%, 79.2%, and 78.4%, respectively, and the mean average precision (mAP) is 1.7% higher than that of the traditional Faster R-CNN model.

## 1. Introduction

With the continuous development of image processing technology, image recognition and target detection technology have been widely used [1,2]. The increasing number of vehicles force researchers to develop new accurate methods for traffic monitoring, accident detection in difficult areas, and parking lot occupation. Therefore, vehicle target detection has become a key research direction in image recognition and object detection technologies [3].

At present, the most used vehicle-type recognition method is to extract vehicle feature information and classify the vehicle type through image processing technology [4,5,6]. However, since the images taken by different cameras usually have different quality, these images of different quality will also be affected by the interference of lighting conditions, perspective distortion, etc., all of which will add difficulty to the type recognition of vehicles [7,8]. Due to the complex road conditions, the large number of vehicles on the road, and the high similarity between vehicles in the real traffic scene, the research on vehicle-type detection is faced with many difficulties and challenges. This research direction, which includes the combination of image processing, pattern recognition, computer vision, statistics, and other fields, has also attracted extensive attention from scholars around the world [9].

Wang et al. [10] proved that Faster R-CNN provides a good vehicle detection effect in low-altitude UAV-based images. Radovic et al. [11] showed that the trained YOLO can detect vehicles from low altitude and top-view drone images with the appropriate dataset and parameter settings. Tang et al. [12] trained UAV images in the original SSD, YOLOv1, and YOLOv2 structures to detect vehicles. The experimental results showed that the detection precision of YOLOv2 is 77.12%, that of the original SSD is 72.95%, and that of YOLOv1 is 67.99%. Valayapalayam et al. [13] presented an occluded vehicle detection algorithm based on the local connectivity and depth model. Elhoseny et al. [14] proposed a vehicle occlusion detection method based on monocular vision and a vehicle tracking system based on monocular vision. In complex traffic conditions, occlusion between vehicles and between vehicles and non-vehicle objects is very common, which poses significant challenges to existing vehicle recognition methods. At present, vehicle-type recognition methods have three main categories: appearance-based, model-based, and feature-based recognition. The appearance-based recognition methods identify vehicle types by utilizing intrinsic features from vehicle images such as shape, texture, and size. Gu et al. [15] utilized the shape features of vehicles to estimate their types and demonstrated that shape information can improve the recognition of vehicle models but does not account for changes in perspective or lighting conditions. Nevertheless, these methods are greatly influenced by the angle at which the camera captures the vehicles and the positions of vehicles in the images. Model-based recognition methods have enhanced the accuracy of vehicle-type recognition by constructing 3D models for different types of vehicles that encompass unique appearance and structural characteristics. Koller et al. [16] proposed a schematic model consisting of 12 parameters for tracking moving vehicles in road traffic scenes. By adjusting length parameters, various types of vehicles can be obtained from these models. However, due to the increasing number of vehicle types over time, it is impractical to provide accurate models for all vehicles in practical research. The feature-based recognition methods extract the information from vehicle images with local or global invariant features. These methods focus on extracting features where only those with more essential characteristics will achieve better results in the recognition.

With the technological progress in the field of deep learning, the accuracy of feature-based object recognition has been greatly improved, and more models with an excellent recognition effect have emerged, such as SPP-Net [17], Fast R-CNN [18], Faster R-CNN [19,20,21], and YOLO [22]. Li et al. [23] proposed a vehicle recognition method based on and-or graph (AOG) and hybrid image templates (HITs), which decomposed the vehicle target into multiple vehicle parts through top–down and left–right segmentation to reduce the impact of vehicle occlusion. This method effectively solves vehicle pose and shape changes. To address the overfitting problem of small targets such as vehicles in UAV images, Hao et al. [24] proposed a denoising convolutional neural network (DCNN). After fine-tuning, the features extracted by the DCNN are more robust and have higher detection rates. It effectively detects vehicles in complex environments such as residential areas. To reduce the false detection rate of vehicle targets caused by occlusion, Zhang et al. [25] proposed a method for vehicle detection in different traffic scenarios based on an improved YOLO v5 network. This method enhances the network’s perception of small targets using the flipped mosaic algorithm and builds a dataset of vehicle targets collected in different scenarios. The detection model is trained based on the dataset. The experimental results show that the flipped mosaic data augmentation algorithm can improve the accuracy of vehicle detection.

Therefore, aiming to meet the requirements of intelligent transportation for vehicle-type recognition in complex traffic scenarios, this paper proposed a recognition model for cars, SUVs, and vans based on the study of the Faster R-CNN to solve the problem of vehicle-type recognition in images. The selection of three vehicle types is based on China’s passenger vehicles. According to the data of the China Passenger Car Association (CPCA) in 2023, cars, SUVs, and vans account for 47.11%, 47.84%, and 5.03% of the total sales volume of passenger vehicles, respectively. By recognizing the types of vehicle targets and their specific location, our model can be used for vehicle management in intelligent transportation. The remainder of this paper is organized as follows: Section 2 puts forward the improved model of Faster R-CNN; Section 3 presents the multi-layer feature combination for Faster R-CNN; Section 4 provides the method of combining contextual features; Section 5 proposes the optimization of the bounding box; Section 6 comprises the experiment results and the numerical results analysis; finally, Section 7 is the conclusion of this paper.

## 2. Improved Model of Faster R-CNN

Both R-CNN and Fast R-CNN models use Selective Search [26] to generate region proposals. However, the Faster R-CNN model has been improved by introducing the region proposal network (RPN) to generate region proposals. Compared with Selective Search, the RPN shares convolution features with the detection network when generating region proposals, which can effectively reduce the time. The RPN first adopts the basic CNN composed of convolutional layers and pooling layers to extract the basic features of the original image, then extracts the image features in a fixed number of region proposals, and uses the region of interest (RoI) pooling layer to fix the region proposal features of the same size. Finally, the RPN adopts the fully connected layer as the output to obtain the probability of whether the region proposal is the target object. To further optimize the performance of Faster R-CNN in vehicle-type recognition, this paper mainly made the following three improvements.

1. In terms of object recognition with Faster R-CNN, simply increasing the network depth to improve the expression ability of feature information is limited by computational resources and time. Each convolutional layer in the model represents different features of the target objects, but the basic Faster R-CNN model only utilizes the features of the last convolutional layer. This paper combined high-level features and low-level features to fully utilize each layer’s features, thereby improving object recognition accuracy.
2. In the original images, the background information plays a significant role in recognizing objects. For example, vehicles are often found on roads or in open parking lots. By effectively utilizing the background information from entire original images, it is possible to improve the accuracy of recognition models. Contextual features refer to the background information extracted through feature extraction in whole images. The traditional Faster R-CNN model only adopts local features from region proposals and does not leverage contextual features. Therefore, based on the Faster R-CNN model, contextual features were utilized in this paper to optimize vehicle-type recognition.
3. When generating bounding boxes for the object in Faster R-CNN, there are often numerous duplicate bounding boxes surrounding real objects due to a large number of region proposals generated by the RPN. The Faster R-CNN model deletes all generated bounding boxes except for the one with maximum probability. Although this method can effectively remove multiple bounding boxes appearing on the same object simply and efficiently, it leads to lower positional accuracy of object bounding boxes. In this paper, the non-maximum suppression (NMS) algorithm was used to filter the object bounding box, and the information in other bounding boxes with higher probability values was used to improve the accuracy of the final object bounding box generation.

## 3. Multi-Layer Feature Combination

High-level convolutional layer features undergo multiple pooling of the pooling layers in the CNN, and some basic information contained in shallow features such as texture and contour is lost. In order not to lose the specific information, the features of the high-level convolutional layer output and the features of the low-level convolutional layer output can be used to identify the object at the same time, and the results are combined for the recognition of the object. However, such a method requires extensive training on the dataset, which would waste resources and time. Therefore, a multi-layer feature combination method based on Faster R-CNN was proposed in this paper to combine the output features of different convolution layers for improving object recognition accuracy.

Commonly used backbone networks in the Faster R-CNN model include VGG16 and ResNet. In ResNet, multi-layer feature combination refers to combining the output features from both the third convolutional layer and the fourth convolutional layer together. To make sure that the size of the third convolutional layer’s output matches that of the fourth convolutional layer’s output in dimensionality terms, the output of the third convolution layer is pooled by the pooling layer whose convolution kernel size is 3 × 3 and step size is 2. Then, the third and fourth convolutional layers are combined in the concat layer.

**Figure 1:**
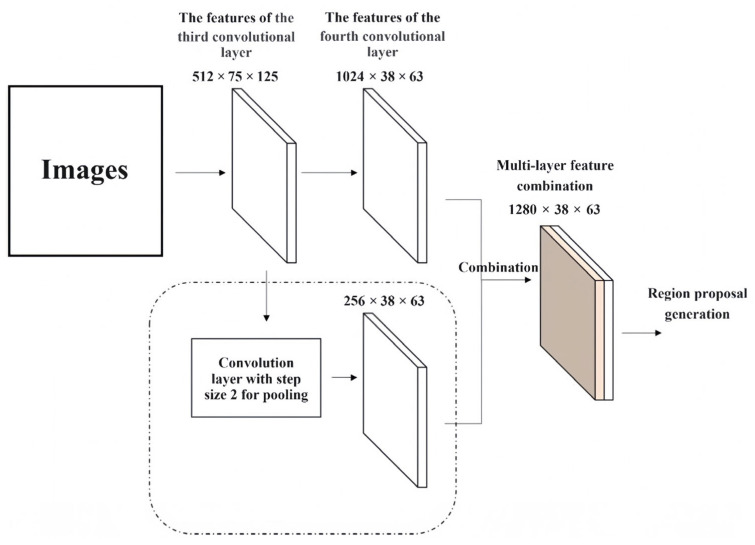
The process of multi-layer feature combination.

Figure 1 shows the process of the multi-layer feature combination proposed in this paper. To improve the expression ability of multi-layer features, this paper used a bottleneck structure [27] to reduce the dimension of output features, which prevents the third convolutional layer from doubling the number of convolution kernels. In this way, the number of parameters introduced are always low, and the number of high-level features are four times those of low-level features. Therefore, the features of the high-level convolutional layer still play a major role in all of the convolutional layer output features.

## 4. The Method of Combining Contextual Features

Context in the field of image recognition refers to the information of the whole image, while contextual features are obtained after the whole image is input into the model for extraction. Different from the local features extracted after the region proposal is generated by the RPN, the contextual features also contain the background information in the original image, and some of the background information can also improve the performance of the recognition. For example, it can generally be recognized that in the image, there are cars on the road, books on the table or shelves, and items such as hats, glasses, and scarves that appear on people’s heads.

Therefore, this paper adopted contextual features in Faster R-CNN to improve the performance of vehicle-type recognition, since the feature extraction of the whole original image is required after the network is improved by the multi-layer feature combination in the previous section. The recognition accuracy of Faster R-CNN can be improved by using the contextual features extracted in the multi-layer feature combination step.

**Figure 2:**
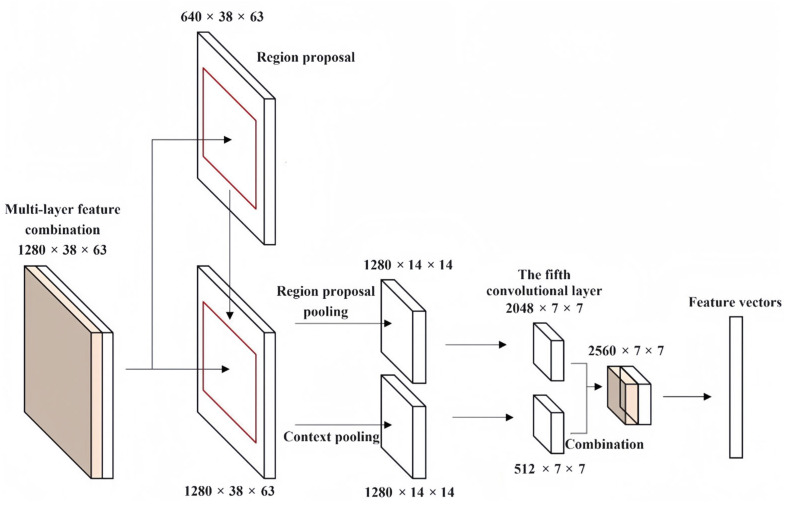
The process of combining contextual features.

To combine the features in the whole original image with the local features of the region proposals, two different sizes of the features need to be converted into the same size. However, the output features of the last convolutional layer have been changed to 1/16 of the original image after four pooling layers in the model. Therefore, the multi-layer feature combination proposed in Section 3 is firstly context pooled and divided into multiple regions with a size of 14 × 14. The fixed-size features are obtained by maximum pooling in all regions, and then, the size of features is reduced to 7 × 7 using a convolution layer whose step size is 2. Finally, after combining them with the local features of region proposals, the final feature vectors are obtained. The process of combining contextual features is shown in Figure 2.

## 5. Optimization of the Bounding Box

**Figure 3:**
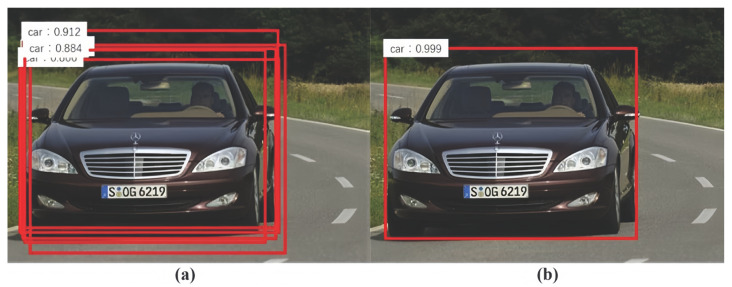
The effect of the NMS algorithm: (**a**) before the NMS algorithm; (**b**) after the NMS algorithm.

After an image is input into the Faster R-CNN model for object recognition, the bounding box of the detected object will be output. To improve the accuracy of recognition, a voting strategy for the bounding box was introduced in this paper. When calculating the output of the object bounding box, Faster R-CNN will select the object bounding box based on the recognition probability of the target object and retain the first 100 bounding boxes with the highest probability at most. However, multiple bounding boxes do not appear on an object, and the NMS algorithm can generally be used to remove repeated bounding boxes for the same object. When the model generates multiple bounding boxes on the same object, it only retains the bounding box whose intersection over union (IoU) is greater than the set threshold and whose recognition probability is the maximum. The effect of the NMS algorithm is shown in Figure 3. All of the repeated bounding boxes in the target object (the black car) in the image are deleted.

Although these deleted object bounding boxes do not represent the maximum object recognition probability, they also contain a lot of object position information in the original image. We can find the position of the object in the image more accurately with the proper use of the position information. This paper introduced a voting algorithm based on object recognition probability. As shown in Figure 3, we adopted the recognition probability of each bounding box for the target object (the black car) as a weight for the voting algorithm to help us obtain more accurate position information of the object in the image. The specific method is to first apply the NMS algorithm with a threshold of 0.3 to the recognition results of a certain type of the object *a*, and then to adopt the bounding box voting algorithm, as shown in Formulas (1) and (2), to obtain more accurate object positions.

(1) $${\tilde{G}}_{a,i}=\frac{\sum_{j:G_{a,j}\in A\left(G_{a,i}\right)}Q_{a,j}\cdot G_{a,j}}{\sum_{j:G_{a,j}\in A\left(G_{a,i}\right)}Q_{a,j}}$$

(2) $$Q_{a,j}=\max\left(0,P_{a,j}\right)$$

where ${\tilde{G}}_{a,i}$ is the newly generated object bounding box of the *i*th object *a*, *A* is the set of threshold values greater than 0.5 in the object bounding box, $P_{a,j}$ is the set of recognition probabilities of the generated object bounding boxes, and $Q_{a,j}$ is the weight of the voting algorithm.

**Figure 4:**
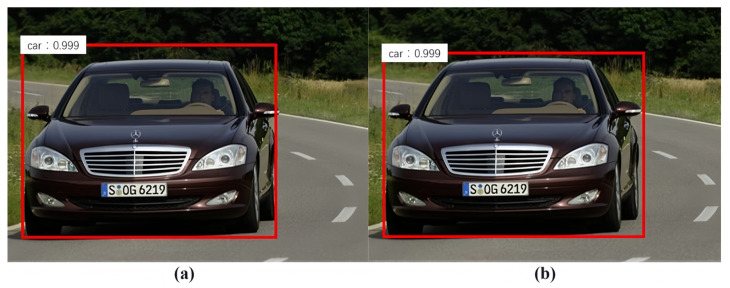
The effect of the voting algorithm: (**a**) before the voting algorithm; (**b**) after the voting algorithm.

Through the calculation, a new bounding box of the target object is obtained. The vehicle-type recognition results before and after our voting algorithm are shown in Figure 4. Compared with the bounding box without the voting algorithm, the position accuracy of the bounding box has been significantly improved.

**Figure 5:**
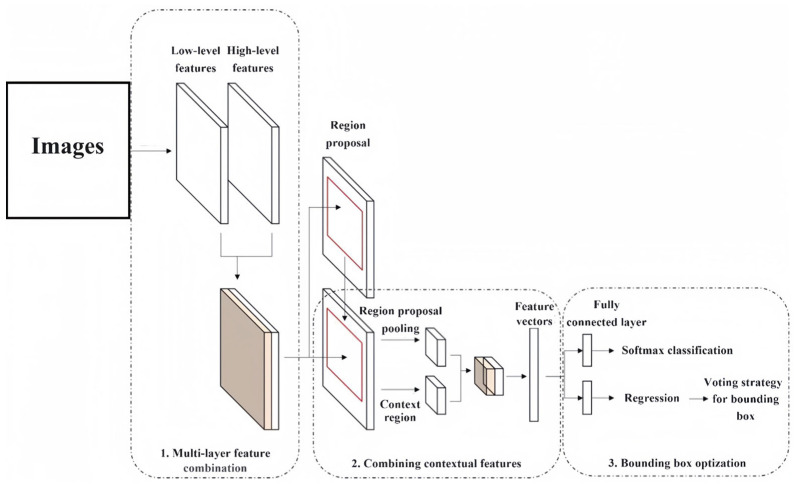
The improved structure of Faster R-CNN.

After the improvement in Faster R-CNN in the three ways proposed in this paper, the overall structure is shown in Figure 5.

## 6. Experiment and Discussion

### 6.1. Experimental Process and Dataset

To validate the performance of our improved Faster R-CNN model, this section will analyze the model from the perspectives of a vehicle dataset, experimental evaluation indicators, and improvement effects. Firstly, if the IoU of the object bounding box *G* generated by Faster R-CNN and the ground truth *G_gt_* marked in the test set satisfy Formula (3), the object is judged to be the target, otherwise, it is judged not to be the target.

(3) $$\mathrm{IoU}\left(G,G_{gt}\right)=\left(G\cap G_{gt}\right)/\left(G\cup G_{gt}\right)\geq T_{r}$$

where *T_r_* is the fixed threshold, and *T_r_* ≥ 0.5 in most experiments. For the ground truth *G_gt_* in an image of size *m* × *n*, the fixed threshold *T_r_* should satisfy Formula (4).

(4) $$T_{r}=\min\left(0.7,\left(m\times n\right)/\left[\left(m+10\right)\times \left(n+10\right)\right]\right)$$

The recall rates of different models are expressed by *rr* and the precision rates are expressed by *pr*, which can be obtained by Equations (5) and (6). Where *TP* stands for true positive and *FP* stands for false positive, they represent the number of bounding boxes that are recognized to be correct and wrong, respectively, and *M_a_* is the number of ground truths in the images for the vehicle type *a*.

(5) $$rr=TP/M_{a}$$

(6) $$pr=TP/\left(TP+FP\right)=\left(recall\cdot M_{a}\right)/\left(recall\cdot M_{a}+FP\right)$$

Then, the AP of each type is calculated by taking 11 positions on the range of recall rates [0, 1] at intervals of 0.1, and converting the precision rates of the vehicle type into a piecewise function of the corresponding recall rates. The AP of the vehicle types is obtained by calculating the area contained in the function curve. Finally, the mAP of the whole test set is the mean value of the vehicle type AP.

**Figure 6:**
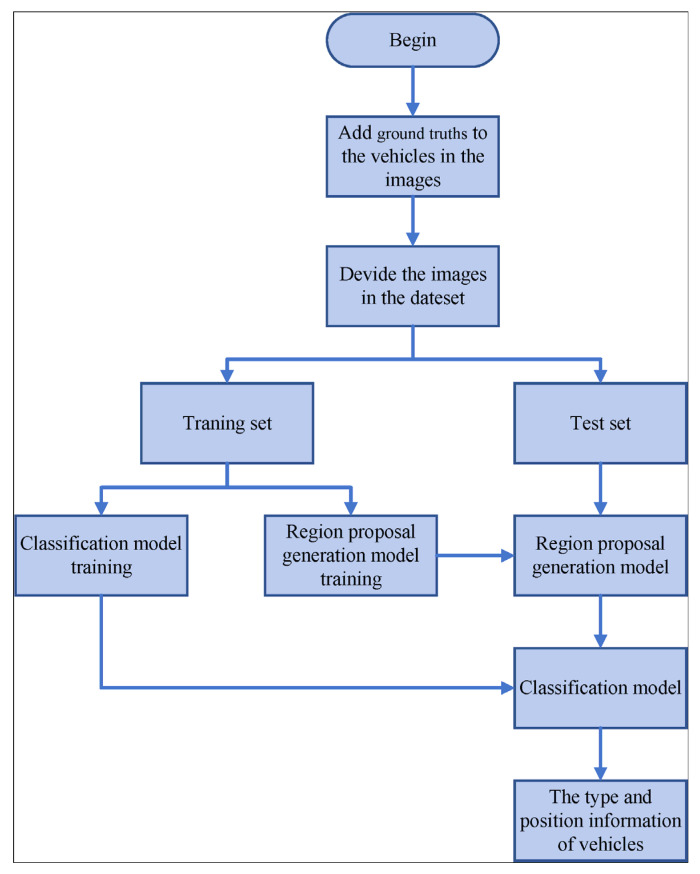
The process of model training and testing.

In this paper, the MIT DriveSeg Dataset [28], the Caltech Pedestrian Detection Benchmark [29], and 1000 vehicle images on the internet were used as the dataset, mainly including a total of three types of vehicle samples (including cars, SUVs, and vans). The MIT DriveSeg Dataset and the Caltech Pedestrian Detection Benchmark are often used as the basic dataset for some vehicle recognition methods and have good performance in recognition results. The MIT DriveSeg dataset is a large-scale traffic scene segmentation dataset that annotates road objects at the pixel level across 5000 video frames. The images in the dataset contain information about various vehicles on city streets. The Caltech dataset consists of approximately 10 h of video taken from vehicles traveling through regular traffic in an urban environment, and the images in the dataset contain a variety of vehicles traveling on urban roads. We used a total of 12,000 images as the training set and a total of 1916 images of three types of vehicles as the test set. The process of model training and testing is shown in Figure 6. Firstly, ground truths are manually added to various vehicles in the dataset, and the marked images are divided into the training set and test set. The training set is used to train the classification model and the region proposal generation model, and the test set is used to test the recognition effect. During the experiment, region proposals of vehicle types are extracted from the original image, and then, the type and position information of different vehicles in the whole image are obtained.

### 6.2. Experimental Results and Analysis for the Recognition Accuracy

In the Faster R-CNN model, region proposals need to be generated first, that is, regions where the object may appear in the original image are found, and then, local features are extracted. Therefore, to improve the accuracy of object recognition, an effective solution is to improve the quality of the generated region proposals. Thus, to include all vehicle types when the number of generating region proposals is as small as possible, the recall rates can be improved without increasing the number of generated region proposals. In this paper, the output features of multiple convolutional layers were combined to obtain multi-layer features, and then, the multi-layer features were used as the input of the Faster R-CNN model to improve the generation quality of region proposals. We utilized two experimental groups to test whether the multi-layer feature combination method can improve the quality of generating region proposals. The images in the dataset were used as the original samples to generate region proposals. The two experimental groups calculated the recall rates of generating region proposals under different IoU values. The difference is that the first experimental group generated 2000 region proposals for each training image, while the second experimental group generated only 300 region proposals.

**Figure 7:**
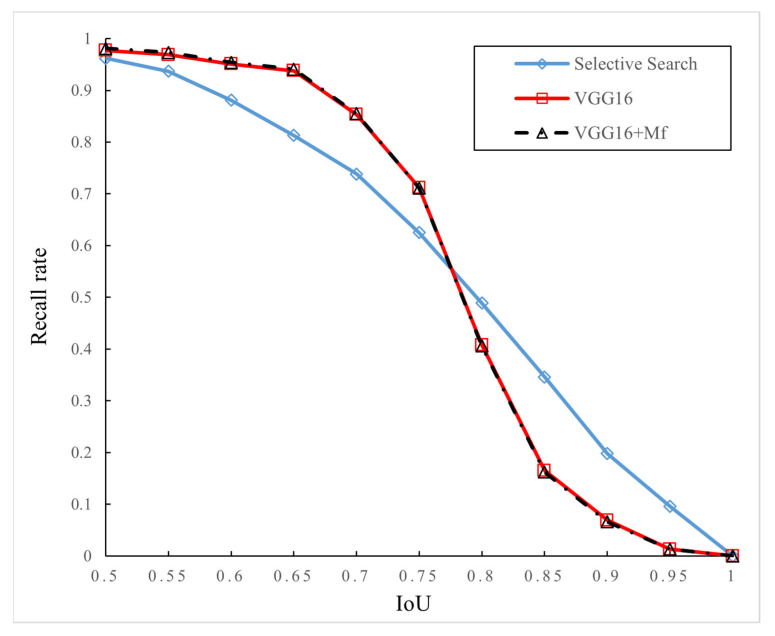
Relationship between recall rates and IoU (2000 region proposals).

In this experiment, Selective Search, the traditional VGG16 network with the RPN (denoted as VGG16), and an improved VGG16 network with the RPN and multi-layer features (denoted as VGG16 + M_f_) are used to generate and analyze region proposals of images. In the case of generating 2000 region proposals, the experimental results are shown in Figure 7. When IoU is 0.5, the three models have relatively good recall rates, all of which are above 0.95. Table 1 records the recall rates when 2000 region proposals are generated with IoU values of 0.5, 0.7, and 0.9, respectively. As can be seen from Table 1, in the case of the same IoU, the improved model based on multi-layer feature combination has a certain degree of improvement in recall rates compared with the traditional VGG16 network.

**Figure 8:**
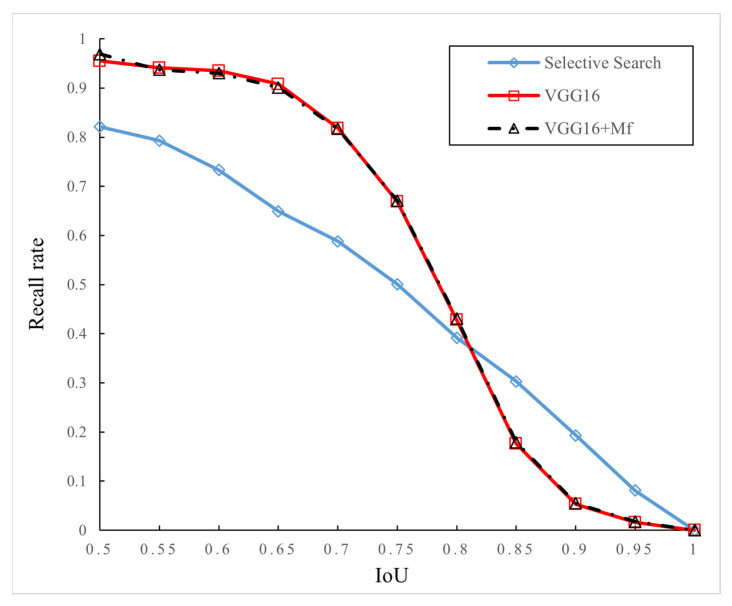
Relationship between recall rates and IoU (300 region proposals).

Then, the number of generated region proposals are reduced from 2000 to 300, and other parameters remain unchanged for the second experimental group. The experimental results are shown in Figure 8. It can be seen from the figure that the Selective Search greatly reduces the recall rates due to the reduction in the number of generated region proposals, which also greatly affects the recognition performance. However, the other two models based on the VGG16 network are less affected by the number of generated region proposals. Table 2 records the recall rates when 300 region proposals are generated with IoU values of 0.5, 0.7, and 0.9, respectively. As can be seen from the table, compared with the traditional VGG16 network, the improved model based on the multi-layer feature combination proposed in this paper has a certain degree of improvement in the recall rates, and the improvement amplitude is greater than that in the case of 2000 region proposals.

To further verify the efficiency of multi-layer feature combination in the Faster R-CNN model, we adopted the ResNet50 network to design the following three comparative experimental groups. The first group is trained and tested using the original ResNet50 network (denoted as R_0_). The second and third groups add the multi-layer feature combination in the ResNet50 network. In the second group, the features of the third convolutional layer output are combined with those of the fourth convolutional layer output after maximum pooling (denoted as P_1_). The third group adopts the convolutional layer whose convolution kernel size is 3 × 3 and step size is 2 for pooling operations (denoted as P_2_). The final results are shown in Table 3. It can be seen from the table that the different multi-layer feature combination methods also have an impact on the recognition AP. The mAP of the second experimental group is increased by 0.29% compared with the first experimental group, and the mAP of the third experimental group is increased by 0.70% compared with the first experimental group.

Then, based on the original Faster R-CNN (denoted as O_1_) model, the three models with the backbone networks VGG16, ResNet50, and ResNet101 are trained and tested by adding the multi-layer feature combination used in model P_2_ (denoted as M_1_). The results are shown in Table 4. In the VGG16 network, the mAP of vehicle-type recognition is increased by 0.57% after adopting the multi-layer feature combination. In the ResNet50 network, the mAP of vehicle-type recognition is increased by 0.71% after adopting the multi-layer feature combination. In the ResNet101 network, the mAP of vehicle-type recognition is increased by 0.61% after adopting the multi-layer feature combination. The improvement in mAP proved that the multi-layer feature combination proposed in this paper can improve the vehicle-type recognition precision of the Faster R-CNN model.

Faster R-CNN with the ResNet50 network is used as the basic model for this experimental comparison. The first experimental group adopts the Faster R-CNN model with the ResNet50 network (denoted as O_2_) to train and test the dataset. The second experimental group adds the contextual features based on the first experimental group (denoted as N_1_), and the third experimental group adds the bounding box optimization based on the second experimental group (denoted as N_2_). The AP of vehicle-type recognition in the three experimental groups is shown in Table 5. It can be concluded that mAP is increased by 0.31% after adding the contextual features, and the mAP is increased by 0.50% after adding the contextual features and the bounding box optimization.

**Figure 9:**
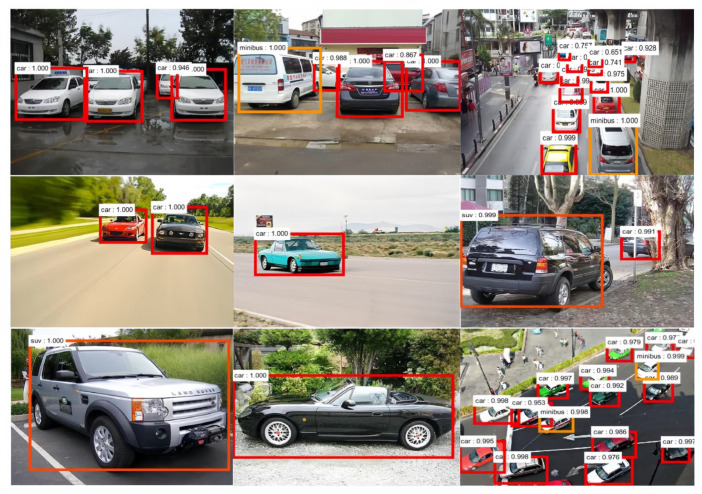
The recognition results of the improved Faster R-CNN model.

Finally, based on the multi-layer feature combination (M_1_), we added the contextual features and bounding box optimization to the Faster R-CNN model for experiments (denoted as M_2_); the results of the vehicle-type recognition tests are shown in Table 6. Compared with the results in Table 4, the mAP of the VGG16 network is increased by 0.73% after adding multi-layer feature combination, contextual features, and object bounding box optimization (compare M_2_ + VGG16 with O_1_ + VGG16), and the mAP is increased by 0.16% after contextual feature combination and bounding box optimization (compare M_2_ + VGG16 with M_1_ + VGG16). The mAP of the ResNet50 network is increased by 0.98% (compare M_2_ + ResNet50 with O_1_ + ResNet50), and the mAP is increased by 0.27% (compare M_2_ + ResNet50 with M_1_ + ResNet50). The mAP of the ResNet101 network is increased by 1.72% (compare M_2_ + ResNet101 with O_1_ + ResNet101), and the mAP is increased by 1.12% (compare M_2_ + ResNet101 with M_1_+ResNet101). Some of the vehicle-type recognition results of the improved Faster R-CNN model are shown in Figure 9. It can be seen from the figure that the improved model proposed in this paper based on Faster R-CNN can better identify the vehicle types in images of actual complex traffic.

### 6.3. The Time Performance of the Improved Faster R-CNN Model

The previous experiments verified that the improved Faster R-CNN model designed in this paper can enhance the recognition accuracy of vehicle types in images. In addition, we also need to analyze the time performance of the improved model. Thus, a comparison experiment was designed to compare the recognition time of the model before and after the improvement. Based on the original Faster R-CNN model, this paper simulated the recognition time of two models whose backbone networks are VGG16 and ResNet50, respectively. The experimental results are shown in Table 7. The multi-layer features and contextual features adopted in this paper added more feature parameters to the Faster R-CNN model, and the bounding box optimization used in this paper also increased the computational complexity, eventually leading to an increase in the recognition time. As can be seen from the results of Table 7, the improvement in the traditional Faster R-CNN model (VGG16 network to ResNet50 network) increases the average recognition time of each image by more than 60%, and the mAP of vehicle-type recognition is increased by 2.03%. Compared with the original Faster R-CNN model, the improved model adopted in this paper increases the average recognition time of each image by less than 10%, and the mAP of vehicle-type recognition is increased by 0.86%. Therefore, the improved Faster R-CNN model proposed in this paper has a relatively high efficiency for vehicle-type recognition.

## 7. Conclusions

The large increase in the number of vehicles in the city has brought many social problems, and traditional manual vehicle management will consume a lot of time, effort, and material resources; therefore, the use of deep learning for vehicle-type recognition in images has become particularly important in the field of intelligent transportation. Based on the Faster R-CNN model, this paper proposed three ways to improve it, including multi-layer feature combination, the use of contextual features, and bounding box optimization. The improved model was then experimentally analyzed through the created vehicle dataset. The experimental results show that the improved network has a recognition AP of 83.2%, 79.2%, and 78.4% for the three vehicle types in the ResNet101 model, respectively. Compared with the traditional Faster R-CNN method, the mAP is increased by 1.7%. In future work, we will expand the training data sample, such as by using more data for vehicle targets in Microsoft COCO. Meanwhile, due to the introduction of multi-layer features and contextual features in this paper, the recognition time was increased to a certain extent. Therefore, improving the time performance is also an important direction of future research.

## Figures and Tables

**Table 1:** Recall rates under different IoU values (2000 region proposals).

IoU	Selective Search	VGG16	VGG16 + M_f_
0.5	0.962	0.977	0.981
0.7	0.738	0.853	0.859
0.9	0.198	0.069	0.066

**Table 2:** Recall rates under different IoU values (300 region proposals).

IoU	Selective Search	VGG16	VGG16 + M_f_
0.5	0.821	0.955	0.969
0.7	0.588	0.818	0.817
0.9	0.193	0.053	0.055

**Table 3:** The effect on vehicle-type recognition based on the Faster R-CNN model.

Experimental Group	Model	AP (Cars)	AP (SUVs)	AP (Vans)
Group 1	R_0_	81.34%	77.41%	76.48%
Group 2	P_1_	81.63%	77.82%	76.65%
Group 3	P_2_	82.07%	78.11%	77.16%

**Table 4:** The effect of multi-layer feature combination in the Faster R-CNN model on vehicle-type recognition.

Model	Backbone	AP (Cars)	AP (SUVs)	AP (Vans)
O_1_	VGG16	79.25%	75.24%	74.62%
O_1_	ResNet50	81.32%	77.47%	76.40%
O_1_	ResNet101	81.51%	77.52%	76.79%
M_1_	VGG16	79.97%	75.71%	75.14%
M_1_	ResNet50	82.03%	78.12%	77.17%
M_1_	ResNet101	82.10%	78.28%	77.26%

**Table 5:** The enhancement effect on vehicle-type recognition.

Experimental Group	Model	AP (Cars)	AP (SUVs)	AP (Vans)
Group 1	O_2_	81.37%	77.43%	76.45%
Group 2	N_1_	81.61%	77.86%	76.72%
Group 3	N_2_	81.84%	77.97%	76.93%

**Table 6:** The enhancement effect of contextual features and bounding box optimization.

Model	Backbone	AP (Cars)	AP (SUVs)	AP (Vans)
M_2_	VGG16	80.03%	75.97%	75.31%
M_2_	ResNet50	82.33%	78.36%	77.45%
M_2_	ResNet101	83.28%	79.25%	78.46%

**Table 7:** The average recognition time and mAP of the models.

Model	Backbone	Average Recognition Time	mAP
O_1_	VGG16	0.236	76.37%
O_1_	ResNet50	0.381	78.40%
M_2_	VGG16	0.256	77.10%
M_2_	ResNet50	0.411	79.38%

## Data Availability

All relevant data are within the paper.
